# H3K9 post-translational modifications regulate epiblast/primitive endoderm specification in rabbit blastocysts

**DOI:** 10.1186/s13072-025-00568-8

**Published:** 2025-01-13

**Authors:** Wilhelm Bouchereau, Hong-Thu Pham, Worawalan Samruan, Van-Hong Vu, Thierry Joly, Marielle Afanassieff, Pierre Savatier, Rangsun Parnpai, Nathalie Beaujean

**Affiliations:** 1 Univ Lyon, Université Lyon 1, INSERM, Stem Cell and Brain Research Institute U1208, INRAE USC 1361, Bron, F-69500 France; 2 Embryo Technology and Stem Cell Research Center, School of Biotechnology, Institute of Agricultural Technology, Suranaree University of Technology, Nakhon Ratchasima, 30000 Thailand; 3 Université de Lyon, VetAgro Sup, Interactions Cellules Environnement (ICE), Marcy l’Etoile, 69280 France; 4 ISARA Lyon Agrapole, 23 rue Jean Baldassini, Lyon Cedex 07, 69364, France

**Keywords:** H3K9 post-translational modifications, Embryo, Epiblast, Endoderm, Rabbit, Histone deacetylase, Histone methyltransferase

## Abstract

Post-translational modifications of histone H3 on lysine 9, specifically acetylation (H3K9ac) and tri-methylation (H3K9me3), play a critical role in regulating chromatin accessibility. However, the role of these modifications in lineage segregation in the mammalian blastocyst remains poorly understood. We demonstrate that di- and tri-methylation marks, H3K9me2 and H3K9me3, decrease during cavitation and expansion of the rabbit blastocyst. Notably, H3K9me3 levels are particularly low in inner cell mass cells at the onset of blastocyst formation but increase again just before gastrulation. Conversely, H3K9ac is abundant in early blastocyst stages but decreases during the transition from the inner cell mass to the epiblast. These distinct distribution patterns correlate with high expression levels of methyltransferases (EHMT1, EHMT2, SETDB1) and deacetylases (HDAC1, HDAC2, HDAC5) in expanding blastocysts. Functionally, inhibiting H3K9me2/3 through an EHMT1/2 inhibitor disrupts primitive endoderm segregation, whereas enhancing histone acetylation (including H3K9ac) using a class I HDAC inhibitor promotes epiblast expansion at the expense of the primitive endoderm. These modifications impact the expression of genes associated with pluripotency and lineage determination, underscoring the importance of H3K9 modifications in embryonic cell fate decisions.

**Supplementary Information:**

The online version contains supplementary material available at 10.1186/s13072-025-00568-8.

## Introduction

Histone H3 modifications play a pivotal role in determining cell fate during development in response to environmental cues. Methylation of lysine 9 on histone H3 (H3K9me) is an epigenetic modification that represses gene expression by facilitating the formation of heterochromatin [1–4]. Conversely, acetylation at the same lysine residue (H3K9ac) enhances nucleosome accessibility to the transcriptional machinery, thereby activating transcription. In mouse embryonic stem cells (mESCs), there is a constitutive elevation of H3K9 acetylation, creating a chromatin environment conducive to gene activation [5, 6]. H3K9 acetylation is also associated with stemness genes, enriched in RNA polymerase III transcription factor C (TFIIIC), indicating a role in regulating pluripotency genes [7].

Post-translational modifications of H3K9, including di-methylation (H3K9me2), tri-methylation (H3K9me3) and acetylation (H3K9ac), undergo dynamic alterations during preimplantation development [8, 9]. Notably, H3K9me3 plays a crucial role in terminating developmental plasticity and establishing higher-order heterochromatin in mouse preimplantation embryos [10]. Upon the formation of the first two lineages –the extraembryonic trophectoderm (TE) and the inner cell mass (ICM)– H3K9me3 mark represses gene promoters (such as *Cdx2*) and is absent from active gene promoters (such as *Oct4* and *Nanog*) in ICM cells [11]. Marks of H3K9me3 at gene promoters become apparent when the ICM segregates into primitive endoderm (PE), which forms the yolk sac, and pluripotent epiblast (EPI), which subsequently gives rise to the three embryonic germ layers [12]. H3K9 di-methylation (H3K9me2) represents an intermediate state in H3K9 methylation, bridging euchromatic and heterochromatic regions [13]. This modification is often localized at the periphery of heterochromatin and is critical for development. Notably, the methyltransferase Ehmt2 (also known as G9a), which mediates mono- and di-methylation of H3K9, is indispensable for proper development. Loss of Ehmt2 leads to severe growth retardation and early lethality in mice, with more pronounced defects in embryonic tissues compared to the TE, underscoring the epigenetic asymmetry between these lineages [14]. Conversely, treatment with Ehmt2 inhibitors enhances embryonic activation following somatic cell nuclear transfer and increases mouse cloning success rates to 14% when combined with the broad-spectrum histone deacetylase (HDAC) inhibitor trichostatin A (TSA) [15]. Collectively, these findings underscore the critical role of balanced histone H3K9 modifications in ensuring proper embryonic development.

The role of H3K9 methylation in cell fate determination is further supported by in vitro studies in mESCs. In mESCs, H3K9me2/3 marks developmental genes poised for activation or repression, depending on the differentiation cues they receive. Loss of H3K9me2 disrupts lineage priming and impairs differentiation potential, highlighting its critical role in balancing pluripotency and lineage commitment [16]. A loss of H3K9me3 at the *Nanog* locus increases Nanog expression, delays commitment to differentiation, and compromises development towards a primitive endoderm fate [7]. Similarly, demethylation of H3K9 by lysine demethylases Kdm3 and Kdm4 enhances the self-renewal and survival of mESCs [17–19]. Acetylation of H3K9 also plays a role in regulating the balance between the naive and primed states of pluripotency. Treatment of murine and human pluripotent stem cells (PSCs) with HDAC inhibitors promotes histone acetylation, thereby facilitating their reprogramming from the primed to the naive state [20–22].

The role of H3K9 modifications in species other than mice has been relatively unexplored. During the preimplantation cleavage stages in rabbits, reorganization of heterochromatin and alterations in H3K9 modifications occur at the 4-cell stage, just before embryonic genome activation (EGA) [23]. This suggests a role for these modifications in early development, similar to that observed in mice. In bovines, both H3K9me3 and H3K27me3 have been observed within gene bodies prior to EGA, with chromatin accessibility being restored during EGA [24]. Comparative studies between human and bovine blastocyst formation have shown that the deposition of H3K9me3 at species-specific transposons in the ICM and TE contributes to species-specific differences in the expression of repetitive elements [24]. Additionally, H3K9me2 has been implicated in regulating transposon activity and maintaining genomic stability, underscoring its multifaceted role during early development [13]. However, the impact of H3K9 modifications during later developmental stages—particularly in the specification of EPI and PE lineages—remain largely unexplored.

To address this knowledge gap, we analyzed the dynamics of H3K9me2/3 and H3K9ac in rabbit embryos during blastocyst formation, from day E3.0 (morula stage) until day E6.6 (early primitive streak stage) of development. Indeed, lagomorph development provides easier access to a broader developmental timeframe compared to rodents and primates, as rabbit embryos do not implant until embryonic day 7 [25, 26]. Additionally, like humans and non-human primates, rabbit embryos exhibit late segregation of EPI, TE and PE lineages, making them a more suitable model for studying human preimplantation development than rodents [27]. We also examined the expression of enzymes responsible for adding or removing H3K9me2/3 and H3K9ac marks (writers and erasers) in the ICM and EPI cells using single-cell RNA sequencing (10x Genomics). Ultimately, we assessed the impact of these epigenetic modifications on cell fate specification by targeting epigenetic modifying enzymes with specific inhibitors during blastocyst formation.

## Methods

### Harvesting and culturing rabbit embryos

All procedures in rabbits were approved by the French ethics committee CELYNE (approval numbers APAFIS #6438 and APAFIS #2180-2015112615371038v2) and COMETHEA (number 45, registered under numbers 12/107 and 15/59). Sexually mature New Zealand white rabbits were injected with follicle-stimulating hormone and gonadotropin-releasing hormone, followed by artificial insemination as previously described [28]. Embryos at Day 3.0, 3.5, 4.0, 5.0, 6.0 and 6.6 post-fertilization were recovered from the uterine horns by flushing with a medium composed of DPBS (Dulbecco’s phosphate buffered saline, Gibco, 14190-094), 10% FBS (fetal bovine serum, Corning, 35-079-) and 1% Penicillin Streptomycin Glutamine (PSG, Gibco, 10378- 016). The embryos were then transferred to RDH medium, which is composed of one-third DMEM-glutamax medium (Gibco, 31966-021), one-third RPMI-glutamax medium (Gibco, 61870-010), and one-third F10-glutamax medium (Gibco, 41550-021), supplemented with 4% bovine serum albumin (BSA, Sigma A3311-10G), and 0.8% taurine (Sigma T8691). The mucin coat and zona pellucida were digested by incubating the embryos in 0.5% Streptomyces griseus protease (Sigma P8811-100MG) for 1 to 2 min at 37 °C, and then they were transferred to RDH medium for further experiments. The embryos were cultured in RDH medium in an incubator at 38 °C and 5% O_2_. Inhibitors A366 (MedChem Express, HY-12583) and UF010 (Tocris, 5588) were re-suspended in DMSO at high concentrations, stored at -20 °C, and diluted in PBS just before use. For each experiment, all embryos, including controls and treated groups, were transferred to new culture dishes containing freshly prepared RDH medium. Inhibitors were then added at the appropriate concentrations (10µM for A366 [29] and 2µM for UF010 [30]) in the “treated” groups. After 24 h of treatment, all embryos were transferred to fresh RDH medium, with or without inhibitors, for an additional 24 h. Following these 48 h of culture (with or without inhibitors), embryos were fixed in 4% paraformaldehyde (PFA) for 20 min at room temperature.

### Immunolabeling and image analysis

Immunolabeling was performed on fixed embryos after three washes in phosphate-buffered saline (PBS). The embryos were permeabilized in PBS-0.5% Triton X100 for 30 min and subsequently incubated in a blocking solution (PBS supplemented with 2% bovine serum albumin) for 1 h at room temperature. The embryos were then incubated with primary antibodies diluted in the blocking solution overnight at 4 °C. After two washes (2 × 15 min) in PBS, the embryos were incubated in secondary antibodies diluted in blocking solution at a dilution of 1:500 for 1 h at room temperature. Finally, the embryos were subjected to several washes in PBS before staining DNA with 4′,6-diamidino-2-phenylindole (DAPI; 0.5 µg/ml) for 10 min at room temperature. Primary and secondary antibodies included: goat anti-SOX17 (Bio-Techne, AF1924); goat anti-SOX2 (Biotechne, AF2018); anti-goat Alexa-Fluor 488 nm (Fisher Scientific, A21467); mouse anti-SOX2 (Ozyme, L1D6A2); mouse anti-H3K9me3 (Abcam, ab8898); mouse anti-H3K9ac (GeneTex, GTX630554) anti-mouse Alexa Fluor 647 nm (Invitogen, A32728); rabbit anti-H3K9ac (Active Motif, 39917); rabbit anti-GATA3 (Abcam, Ab19942); rabbit anti- H3K9me2 (Active Motif, 39753); rabbit H4ac (Active Motif, 39043); rabbit anti-SETDB1 (Abcam, Ab12317); rabbit anti-HDAC2 (Abcam, Ab32117); rabbit anti-EHMT1/2 (Abcam, Ab194299); rabbit anti-H3K27ac (Cell Signaling, mAB8173); anti-rabbit Alexa Fluor 555 nm (Fisher Scientific, 10749004), sheep anti-KDM4A (R&D System, AF6434); anti-sheep Alexa Fluor 647 (Fisher Scientific, A21448). Immunolabeled embryos were observed using a SP5 confocal microscope (Leica SP5) with LAS AF software (Leica Application Suite Advanced Fluorescence, version 2.7.3.9723). Acquisitions were performed using an oil immersion objective (40×/1.25 0.75, PL APO HCX; Leica). The images obtained were analyzed using image J software (version 2.1.0). Nuclei were segmented based on DAPI, SOX2, SOX17 or GATA3 staining in merged 2D confocal images. For each embryo, the number of cells and mean fluorescence intensity per cell were measured. Differences in mean fluorescence intensities and cell counts across embryonic stages or experimental conditions were analyzed using Welch’s t-test, with significance levels defined as follows [31]: *p* < 0.05% (*); *p* < 0.01% (**); *p* < 0.001% (***); *p* < 0.0001% (****).

### Single-cell RNAseq and bioinformatics analysis

10X single-cell RNAseq datasets, including all raw read sequence data and count matrices for each developmental stage, as well as the aggregated count matrix from each stage, were obtained from a previous study [27]. For this study, the dataset was re-analyzed, with a focus on acetylation and methylation-specific enzymes, using the same tools.

### Biomark qPCR analysis in single cells

After 48 h of treatment with various molecules, the ICM and surrounding polar trophectoderm were dissected, dissociated, and the single cells were recovered following treatment with 0.05–0.1% trypsin for 5–10 min at 37 °C. Cells from the mural trophectoderm were also collected as lineage controls. Enzymatic activity was stopped by adding 10% fetal bovine serum (Gibco 11563397). The single cells were then harvested using glass micro-capillaries and transferred into 200 µL Eppendorf tubes containing 5µL of buffer from the CellsDirect™ One-Step qRT-PCR kit (ThermoFisher). The samples were immediately frozen at -80 °C. Isolated single-cell from four embryos were used for the control condition, three for the + A366 condition and three for the + UF010 condition, with 96 cells harvested per condition (in a single experiment). Reverse transcription and specific target pre-amplification were performed on these isolated cells using the SuperScript III/RT Platinum Taq mix provided in the CellsDirect™ One-Step qRT-PCR kit (ThermoFisher), along with the targeted primer pairs. To assess sample quality and facilitate filtering, the expression of the TATA-box binding protein (TBP) was monitored in the pre-amplified 96 samples for each condition. Samples with very high Ct values (> 35) were excluded at this stage. Real-time PCR (qPCR) was then performed using the StepOnePlus real-time PCR system and Fast SYBR^®^ Green Master Mix (Applied Biosystems). A total of 260 samples were validated for further analysis (89 samples for control embryos, 82 for A366-treated embryos, and 89 for UF010-treated embryos). They were analyzed using Universal PCR TaqMan Master Mix (Applied Biosystems), coupled with a DNA Binding Dye Sample Loading Reagent (Fluidigm) and Evagreen (Biotium 31000) in 96.96 Dynamic Arrays on a Biomark System. Each 96.96 array allowed the analysis of 93 genes in individual cells. Primer sequences are provided in Table S1.

For Biomark analysis, samples were processed using the Fluidigm^R^ Real-Time PCR Analysis Software (version 4.1.3) and the Singular™ Analysis Toolset R package (version 3.6.2). Using this toolset, Ct values were converted into log2ex values (Tables S2 and S3) according to the following formulas: Log2ex = LOD – Ct, if Ct is < limit of detection (LOD); and Log2ex = 0, if Ct is > = LOD. The LOD is set to 24 by default, corresponding to the typical Ct value for a single input copy on the Fluidigm chip. Outliers–samples with overall low expression of all target genes–were excluded using the Fluidigm packages. Cell lineage assignment was based on the expression of specific marker genes (epiblast markers: *FGF4*, *GDF3*, *KDM4A*, *KDM4C*, *KLF4*, *KLF17*, *NANOG*, *NODAL*, *POU5F1*, *PRDM14* and *SOX2*; primitive endoderm markers: *CXCR4*, *FOXA2*, *GATA4*, *GATA6*, *OTX2*, *PDGFRA* and *SOX17*; and trophectoderm markers: *CDX2*, *FABP3*, *GATA2*, *GATA3*, *KRT8*, *KRT18*, and *TFAP2C*) [27]. Once assigned, the lineages were analyzed separately. The Seurat R package (version 3.2) was used for scaling, normalization, differential expression analysis, and plot generation, as described in the results section.

### Network topology-based analysis

Gene network analysis of differentially expressed genes (DEGs) between control and treated datasets (A366 or UF010) was conducted using the protein-protein interaction (PPI) network from the BioGRID database (biomedical interaction repository; https://thebiogrid.org). Co-expression modules and genes interactions were identified and visualized using WebGestalt (https://www.webgestalt.org), a user-friendly platform integrating known protein-protein interactions with single-cell gene expression correlations [32]. This approach involves leveraging known interaction networks and overlaying expression-based correlations to enhance biological relevance. Neighboring genes were identified using a ranking system based on network connectivity scores. Specifically, the top 10 neighbors were selected using a probability framework derived from the random walk method, which quantifies the likelihood of traversing the network to reach these genes. In the resulting PPI networks, DEGs are displayed as seed nodes (blue), while their top 10 ranked neighbors are shown in grey. Lines connecting genes highlight statistically significant correlations, even when the connection involves non-DE genes.

## Results

### Dynamics of H3K9 methylation and acetylation in rabbit blastocysts

**Fig. 1:**
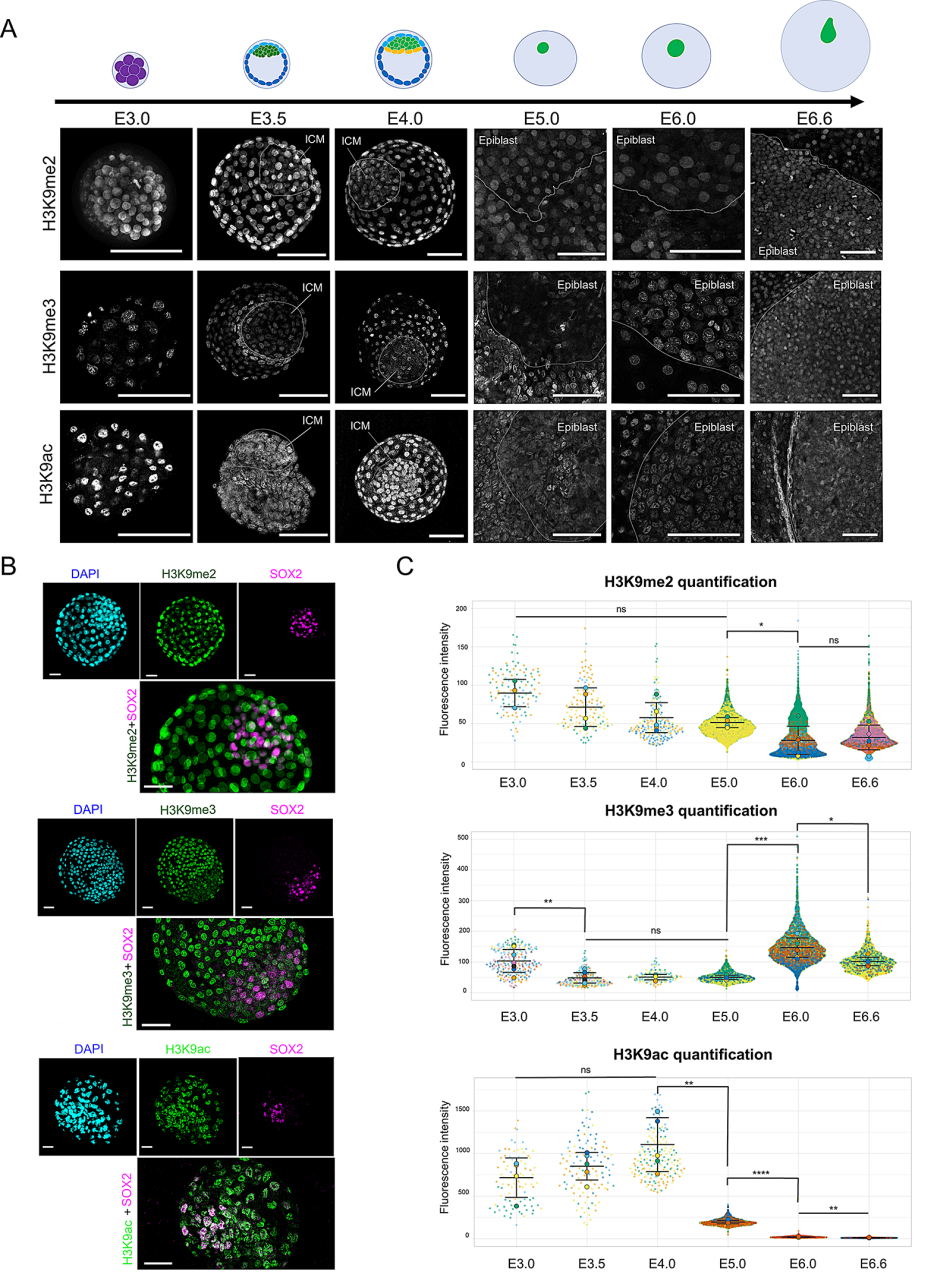
Dynamics of H3K9 methylation and acetylation in rabbit embryos. (**A**) Immunofluorescence imaging of di-methylated (H3K9me2) and tri-methylated (H3K9me3) histone 3 lysine 9, along with acetylated histone 3 lysine 9 (H3K9ac), in embryos at embryonic days E3.0, E3.5, E4.0, E5.0, E6.0, and E6.6. Scale bars: 100 μm. (**B**) Immunofluorescence visualization of SOX2, H3K9me2, H3K9me3, and H3K9ac in E4.0 embryos, with DAPI counterstaining. Scale bars: 40 μm. (**C**) Violin plots representing quantification of fluorescent signals for H3K9me2, H3K9me3, and H3K9ac in SOX2^+^ cells. Each embryo is represented by a unique color. All cells were quantified separately, with the mean of each embryo indicated with a larger dot. *, *p* < 0.05; **, *p* < 0.01; ***, *p* < 0.001; ****, *p* < 0.0001; ns, not significant

To explore the post-translational modifications of H3K9 during early lineage specification, we investigated the patterns of H3K9 di- and tri-methylation, as well as acetylation, in rabbit embryos from the morula stage (embryonic day E3.0) to the expanded blastocyst stage (E6.6). Initially, at the morula stage, all three H3K9 modifications were present in all nuclei (Fig. 1A), but their profiles evolved differently during the cavitation and expansion of the blastocyst (*n* = 10 experiments for both H3K9me3 and H3K9ac, *n* = 3 for H3K9me2; Fig. 1A and Fig. S1). To better track the dynamics of these modifications during cavitation, we performed immunostaining of SOX2, a pluripotency marker specific to ICM/EPI cells, alongside each H3K9 modification (Fig. 1B) and quantified the fluorescent signal in SOX2^+^ cells (*n* = 4 to 10 embryos per stage, Fig. 1C). While H3K9me2 levels progressively decreased from E3.0 to E6.0 in SOX2^+^ cells (Fig. 1B and C), H3K9me3 was more rapidly lost in the ICM of E3.5 embryos and later in the epiblast by day E5.0. On days 3.5-4.0, H3K9me3 immunostaining in SOX2^+^ cells was minimal, indicating low levels of this modification in the pluripotent cells of the early blastocyst (*n* = 3 to 7 embryos per stage; Fig. 1B and C). This mark reappeared in the epiblast by days E6.0 and E6.6, shortly before gastrulation.

Regarding H3K9 acetylation, intense labeling of H3K9ac was observed during the early stages (E3.0/E3.5/E4.0), although labeling intensity decreased from E5.0 onwards (Fig. 1A and Fig. S1). Quantification of H3K9ac in SOX2^+^ cells confirmed the presence of H3K9 acetylation in the pluripotent cells of the blastocyst at E3.5 and E4.0 (*n* = 4 to 6 embryos per stage, Fig. 1B and C).

**Fig. 2:**
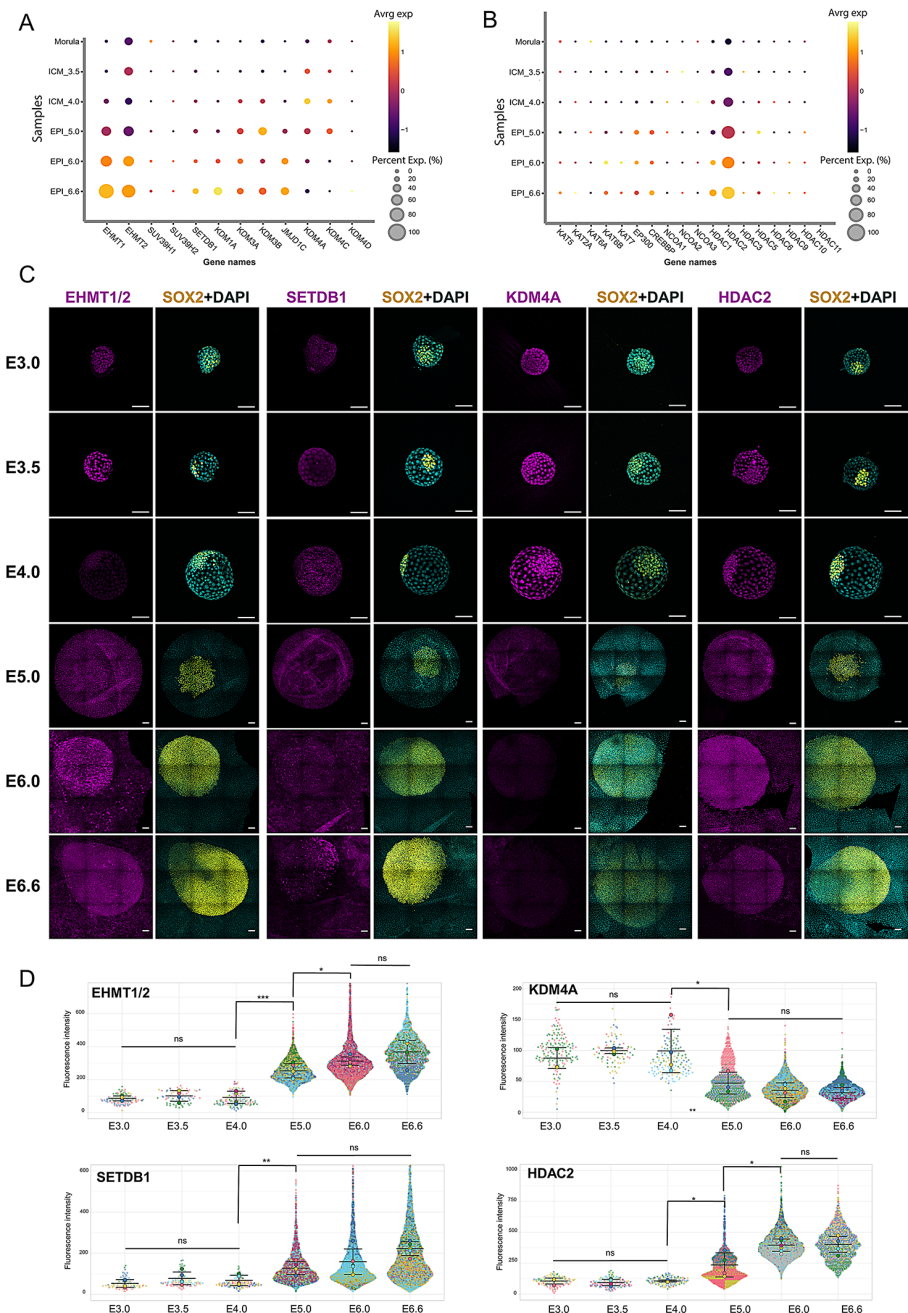
Expression patterns of H3K9 modifying enzymes in single-cell RNA-seq data. (**A-B**) Expression patterns of enzymes responsible for H3K9 methylation (**A**) and acetylation (**B**) in cells of the morula (day 3.0), ICM (day 3.5-4.0), and epiblast (day 5.0-6.0-6.6) cells. The dot size represents the percentage of cells expressing the gene of interest, while the color intensity reflects the average expression level across all cells. (**C**) Immunofluorescence imaging of EHMT1/2, SETDB1, KDM4AA, and HDAC2 in embryos at embryonic days E3.0, E3.5, E4.0, E5.0, E6.0, and E6.6, with DAPI counterstaining and SOX2 co-labeling. *N* = 3 experiments for each enzyme tested. Scale bars: 100 μm. (**D**) Violin plots representing the quantification of the fluorescent signal for each histone-modifying enzyme in SOX2^+^ cells. Each embryo is represented by a unique color, with *n* = 4 to 8 embryos per stage. All cells were quantified separately, with the mean of each embryo indicated with a larger dot. *, *p* < 0.05; **, *p* < 0.01; ***, *p* < 0.001; ns, not significant

We next investigated the expression patterns of H3K9 modifying enzymes using single-cell RNA-seq data (data from [27] were obtained upon manual dissociation of embryos; *n* > 100 for E3.0 and E3.5, *n* ~ 80 for E4.0 and E5.0, *n* ~ 30 for E6.0 and E6.6). During the maturation of the ICM and epiblast, low levels of H3K9 methyltransferases, specifically *EHMT1/2* and *SETDB1*, were observed in the ICM of E3.0 to E4.0; followed by their upregulation in the epiblast from E5.0 to E6.6 (Fig. 2A). This was confirmed by immunostaining and fluorescence quantification in the ICM/EPI cells of embryos, from E3.0 to E6.6, with antibodies recognizing EHMT1/2 and SETDB1 (Fig. 2C and D). Notably, there were minimal variations in the expression of histone demethylases, including *JMJD1*, *KDM1*, *KDM3*, and *KDM4C* at early blastocyst stages when demethylation occurs. However, *KDM4A* was expressed in the ICM at E3.5-E4.0 (Fig. 2A). Immunostaining for KDM4A confirmed higher protein levels at early blastocyst stages (Fig. 2C and D).

On the other hand, the pattern of H3K9ac throughout blastocyst development correlates with the expression of regulatory enzymes, including relatively low levels of histone acetyl transferases (HATs) such as *KAT6B*, *KAT7*, *EP300*, and *CREBBP*, along with increased expression of *HDACs 1* and *5* upon blastocyst expansion (Fig. 2B). In particular, an increase in HDAC2 protein expression in EPI from E5.0 onwards correlates with the decrease in H3K9ac levels (Fig. 2C and D). In summary, these findings indicate that the pluripotent cells of the ICM and early epiblast maintain a consistent level of H3K9 acetylation, accompanied by low levels of H3K9me3 during the early stages of blastocyst development.

### Impact of EHMT1/2 and HDAC class I inhibition on blastocyst development

**Fig. 3:**
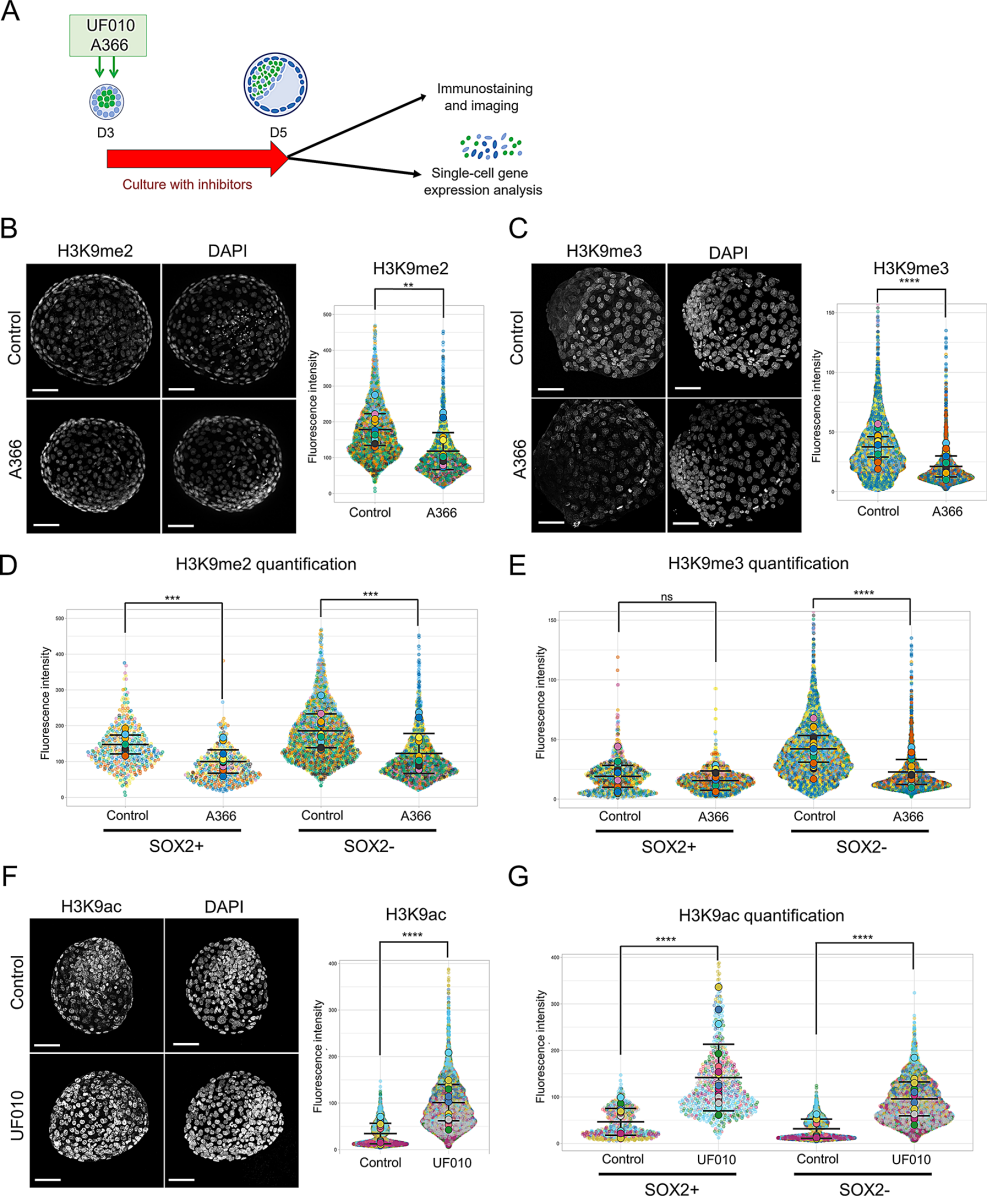
Epigenetic modulation in rabbit embryos post-blastocyst formation. (**A**) Schematic diagram of the experimental approach, detailing inhibitor treatment and subsequent analyses. (**B-C**) Immunolabeling of the H3K9me2 (**B**) and H3K9me3 (**C**) marks, as well as the H3K9ac mark (**C**), in cultured rabbit embryos, treated with and without the A366 inhibitor. DNA was counterstained with DAPI. The fluorescence intensity for each mark was quantified in all cells of the embryos. Scale bars: 100 μm. (**D-E**) Quantification of H3K9me2 and H3K9me3 marks in SOX2^+^ versus SOX2^−^ cell populations of A366-treated and control embryos. (**F-G**) Immunolabeling for the H3K9ac mark (**F**) in cultured rabbit embryos, treated with and without the UF010 inhibitor. DNA was counterstained with DAPI. Scale bars: 50 μm. The fluorescence intensity for each mark was quantified either across all embryos (**F**) or in SOX2^+^ versus SOX2^−^ cell populations of UF010-treated and control embryos (**G**). In the violin plots, each color represents a different embryo, with the mean of each embryo indicated as a larger dot. **, *p* < 0.01; ***, *p* < 0.001; ****, *p* < 0.0001; ns, not significant

To assess the effects of HDAC class I and EHMT1/2 inhibition on blastocyst development, we utilized two specific inhibitors: A366, targeting EHMT1 and EHMT2 enzymes to reduce H3K9 methylation [29, 33]; and UF010, which inhibits class I HDACs (HDAC1, 2, 3 and 8), resulting in increased acetylation of histones [30]. Rabbit embryos obtained after natural fertilization were recovered at day 3.0 and cultured with these inhibitors (10µM for A366 [29] and 2µM for UF010 [30]) for 48 h prior to analysis. Our initial experiments showed that developmental progression to the blastocyst stage was similar across groups, with 98% of control embryos (*n* = 45), 92% of UF010-treated embryos (*n* = 24), and 90% of A366-treated embryos (*n* = 20) reaching this stage, indicating no significant differences (*p* > 0.5). Blastocysts were either fixed in paraformaldehyde for whole-mount immunofluorescent analysis or dissociated into single cells for gene expression analysis using Biomark RT-qPCR (Fig. 3A). Examination of histone marks during the critical period of EPI and PE specification revealed that A366 treatment led to a modest reduction in H3K9me2 and a stronger reduction in H3K9me3 intensities (*n* = 4 or 5 experiments per histone modification tested; Fig. 3B and C). Co-staining with SOX2 to distinguish pluripotent cells (SOX2^+^ vs. SOX2^−^ cells) revealed that in SOX2^−^ cells, A366 treatment reduced both H3K9me3 and H3K9me2 intensities, while in SOX2^+^ cells, only the reduction in H3K9me2 intensity was statistically significant (*n* = 42 embryos with inhibitor and *n* = 46 embryos without inhibitor; Fig. 3D and E and S2). In contrast, UF010 treatment significantly enhanced H3K9ac levels (*n* = 50 embryos with and *n* = 46 embryos without inhibitor; Fig. 3F), as well as acetylation of other histones (H3K27ac and H4ac, Fig S3). Notably, UF010 treatment increased H3K9ac in both SOX2^+^ and SOX2^−^ cells (*n* = 50 and *n* = 46 embryos in ± inhibitor conditions; Fig. 3G).

**Fig. 4:**
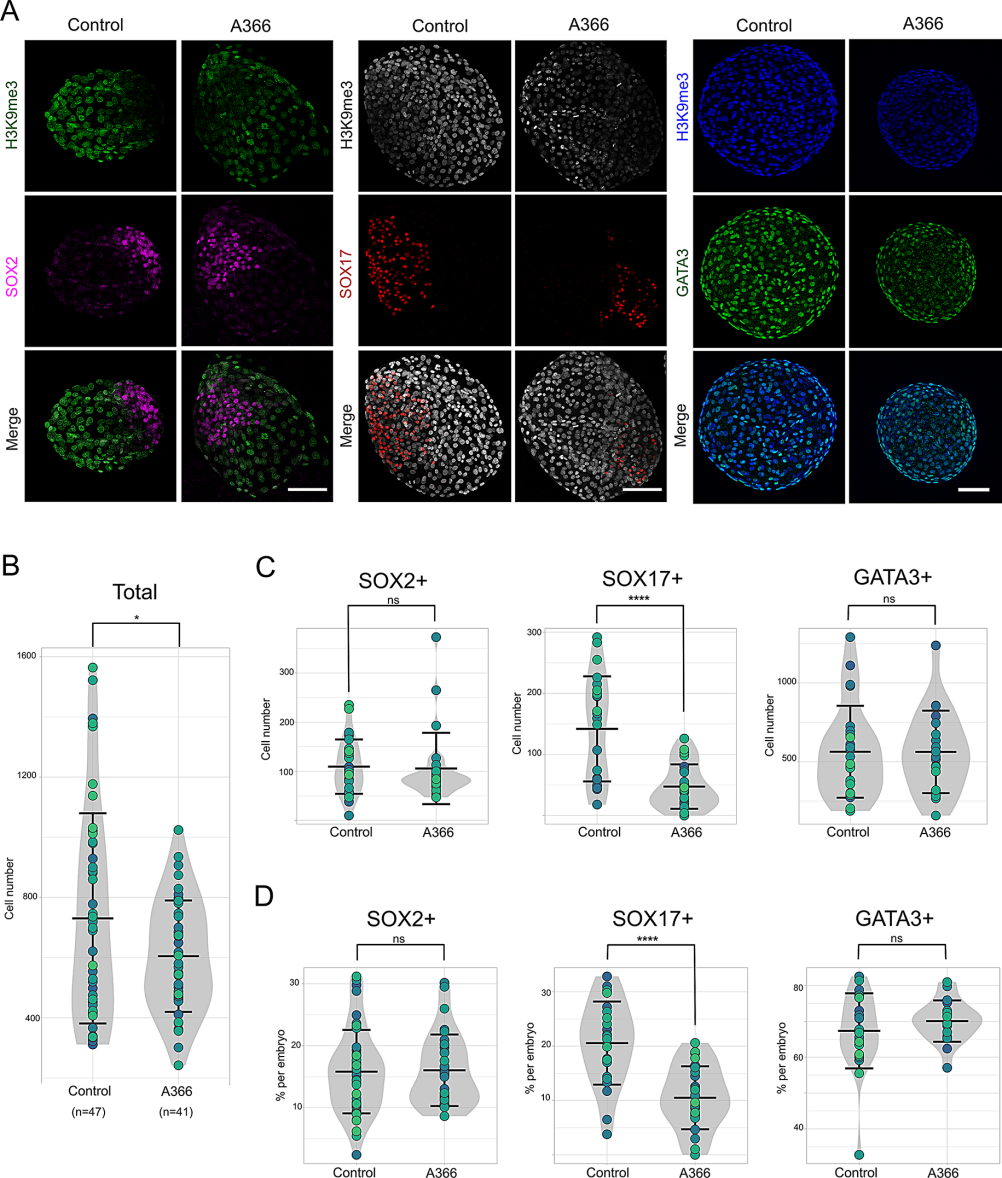
Impact of EHMT1/2 inhibition on cell lineage distribution in rabbit embryos. (**A**) Immunolabeling for the H3K9me3 mark along with SOX2, SOX17 or GATA3 in rabbit embryos treated with A366. (**B-D**) Quantification of total cell count (**B**), SOX2^+^, SOX17^+^, and GATA3^+^ cells per embryo (**C**), as well as their respective percentages within each embryo (**D**). Scale bars: 100 μm. Violin plots represent the distribution of all analyzed embryos, with each embryo identified by a larger dot. *, *p* < 0.05; ****, *p* < 0.0001; ns, not significant. Data were obtained from 4 to 5 different experiments for each combination of antibodies (H3K9me3 + SOX2, H3K9me3 + SOX17, and H3K9me3 + GATA3), including both treated and control embryos

To assess the impact of A366 and UF010 on blastocyst development, total cell count (via DAPI nuclear staining), and the number of SOX2^+^, SOX17^+^, and GATA3^+^ cells per embryo were quantified for each condition to determine the number of EPI, PE and TE cells, respectively (Fig. 4 and S2). Embryos treated with A366 were smaller in size compared to controls (untreated embryos), with a significantly lower total cell count (*n* = 41 and *n* = 47 embryos for treated and control groups, respectively) and displayed a marked reduction in SOX17^+^ cells (~ 47 vs. 142 cells for treated vs. control embryos, *n* = 26 and *n* = 23, respectively) (Fig. 4B and C and D). The number of SOX2^+^ and GATA3^+^ cells remained comparable across groups (*n* = 24 and *n* = 27 embryos for SOX2 groups ± inhibitor; *n* = 23 and *n* = 32 embryos for GATA3 groups ± inhibitor; Fig. 4C and D ans S2). These findings suggest that A366 treatment adversely affects embryonic development, particularly influencing PE formation, as indicated by the reduced proportion of SOX17^+^ cells in A366-treated embryos (11% vs. 22%) and the lower levels of H3K9me2/3 marks in the SOX2^−^ cells.

**Fig. 5:**
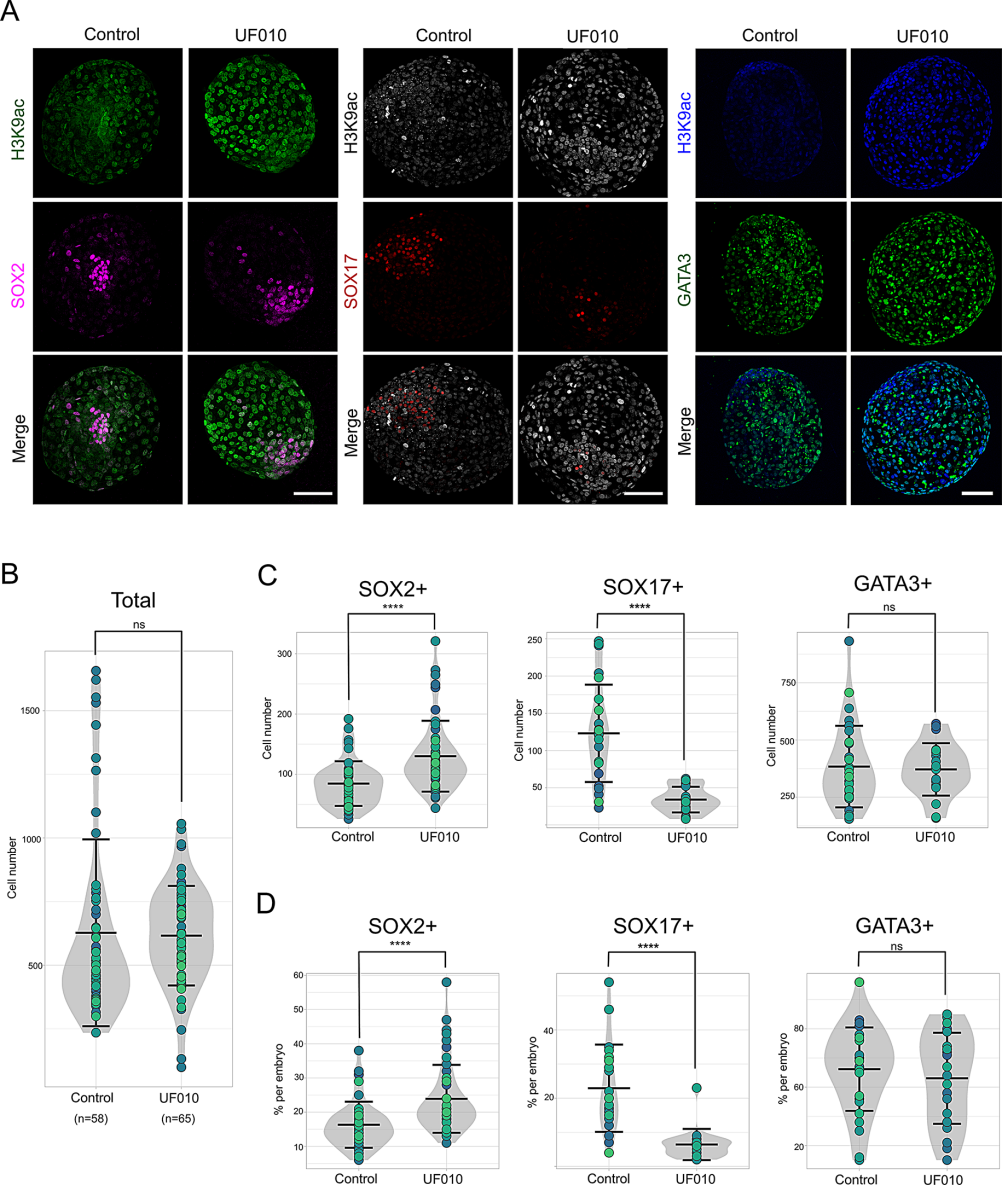
Impact of HDAC class I inhibition on cell lineage distribution in rabbit embryos. (**A**) Immunolabeling for the H3K9ac mark along with SOX2, SOX17, or GATA3 in rabbit embryos treated with UF010. (**B**-**D**) Quantification of total cell counts (**B**), and number of SOX2^+^, SOX17^+^ and GATA3^+^ cells per embryo (**C**), with the respective percentage of each cell type calculated for each embryo (**D**). Scale bars: 100 μm. Violin plots represent all analyzed embryos and their distribution, with each embryo identified by a larger dot. ****, *p* < 0.0001; ns, not significant). Four to five different experiments were performed for each combination of antibodies (H3K9ac + SOX2, H3K9ac + SOX17, and H3K9ac + GATA3), always including both conditions, with or without UF010

Conversely, no significant difference in total cell count was observed between UF010-treated and untreated embryos (*n* = 65 and *n* = 58, respectively; Fig. 5A and B). However, there was a noticeable increase in the number of SOX2^+^ cells in UF010-treated embryos (~ 130 cells vs. 85 cells for treated vs. control embryos; *n* = 50 and *n* = 46, respectively), indicating a positive effect of UF010 on epiblast cell expansion (Fig. 5C and D). This was accompanied by a significant decrease in the number of SOX17^+^ cells (~ 34 cells vs. 123 cells for UF010-treated vs. control embryos; *n* = 19 and *n* = 25, respectively), suggesting that UF010 treatment shifts the balance towards EPI cell proliferation at the expense of PE cells (Fig. 5C and D). No significant difference in TE cell count was observed between UF010-treated and untreated embryos, as shown by GATA3^+^ staining (*n* = 22 and *n* = 29 embryos, respectively; Fig. 5C and D and S3). In summary, our results indicate that inhibition of EHMT1/2 disrupts PE formation, while inhibition of HDAC class I promotes EPI cell expansion, highlighting the critical role of these histone modifications in early embryonic cell fate decisions.

### Impact of EHMT1/2 inhibition on gene expression in EPI, PE and TE lineages

**Fig. 6:**
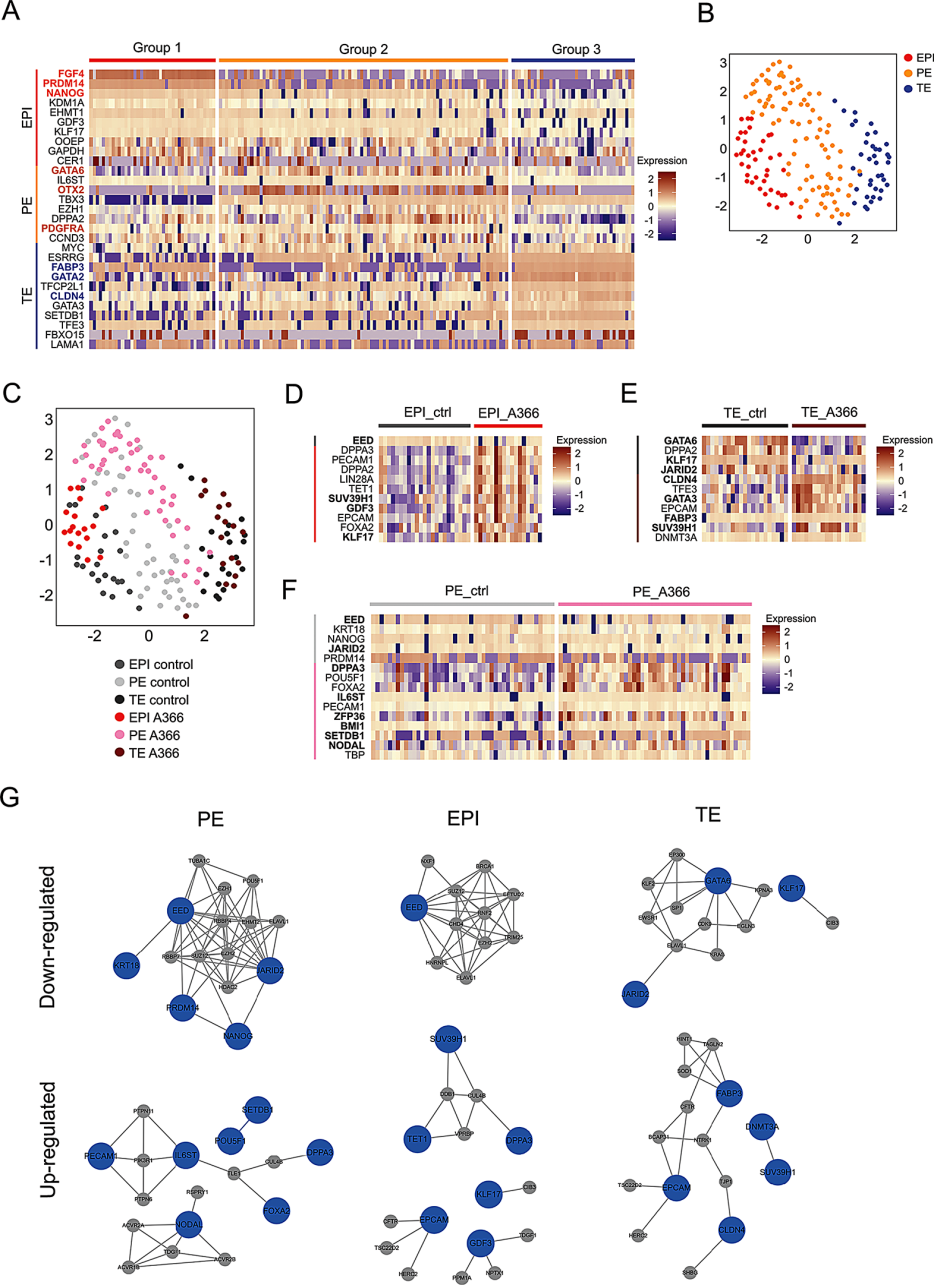
Transcriptomic alterations following EHMT1/2 inhibition in rabbit embryos. (**A**) Heatmap representation of differentially expressed genes among EPI, PE and TE groups. (**B**) Two-dimensional UMAP representation of samples categorized by embryonic lineage. (**C**) Two-dimensional UMAP distinguishing samples based on both lineage and culture conditions. (**D-F**) Heatmap of differentially expressed genes between control and A366-treated samples over 48 h, specifically within the EPI (**D**), TE (**E**), and PE (**F**) lineages. In panels **A**,** D**, **E** and **F**, expression is shown in log2Ex scaled and log-normalized format. (**G**) The protein-protein interaction (PPI) network displays differentially expressed genes (DEGs) as seed nodes in blue and their top 10 ranking neighbors in grey for each cell lineage. Statistically significant correlations between DEGs are depicted as edges (grey lines). This analysis was conducted separately for downregulated and upregulated genes

Single-cell expression analysis of selected genes was performed using Biomark RT-qPCR on embryos treated with A366 and untreated control embryos (Fig. S4). After data filtration and quality control checks (Fig. S4), datasets from control samples (*n* = 89) were compared with those from A366-treated samples (*n* = 82). We used Seurat for integrating the data and K-means graphical clustering to categorize the samples into three groups. These groups corresponded to the three primary embryonic lineages, identified by their expression of lineage-specific genes: *FGF4*, *PRDM14*, and *NANOG* for EPI; *GATA6*, *OTX2*, and *PDGFRA* for PE; and *FABP3*, *GATA2*, and *CLDN4* for TE (Fig. 6A). We then applied UMAP dimension reduction to visualize the data, and identified the three clusters representing the EPI, PE, and TE, respectively (Fig. 6B and S4). When we overlaid the origin of the cells–from A366-treated or control embryos–on the UMAP plot, we observed a segregation between the A366-treated and control cells within the EPI and PE clusters (Fig. 6C). Further analysis showed that A366-treated EPI cells had increased expression of naive pluripotency markers *KLF17* and *GDF3*, and the H3K9 methyltransferase SUV39H1 (Fig. 6D and S5). In the TE lineage of A366-treated samples, there was upregulation of TE-specific genes such as *CLDN4*, *GATA3*, and *FABP3*, along with *SUV39H1*. Conversely, in these cells, the levels of *JARID2*, *GATA6*, and the epiblast marker *KLF17* were lower compared to control TE cells (Fig. 6E and S5). In PE cells from A366-treated embryos, we detected an upregulation of genes associated with naive pluripotency, including *DPPA3*, *IL6ST*, *ZFP36*, and *NODAL*, as well as the H3K9 methyltransferase *SETDB1* and the PRC1 subunit *BMI1*. In contrast, expression of the PRC2 genes *EED* and *JARID2* was reduced (Fig. 6F and S5). These findings suggest that A366 treatment influences gene expression differentially across the three embryonic lineages, potentially altering developmental trajectories. Notably, A366 induced the expression of naive pluripotency markers in both EPI and PE. This finding is consistent with our previous observation that A366 treatment adversely affects PE formation.

We used protein-protein interaction (PPI) network analysis to examine relationships between gene products and identify hub genes. Specifically, we created neighbor-enriched PPI networks for each cell type (EPI, PE and TE) treated with A366, focusing on differentially expressed genes (DEGs) (Fig. 6G). For genes with decreased expression, the PRC2 genes *EED* and *JARID2* were central hub genes connected to all other genes in the networks calculated for the PE and EPI. In contrast, with the TE cells, *GATA6* was the primary hub gene, with a direct connection only to *JARID2*. For genes with increased expression, the networks exhibited greater variability. In the PE, *IL6ST* stood out as a hub gene with connections to *PIK3R1*, *PTPN6*, and *PTPN11*, all of which play a role in the JAK-STAT3 signaling pathway. *NODAL*, important in the activin receptor signaling pathway, was another hub gene in this network. In EPI cells, we observed several small clusters of upregulated genes, but no single gene dominated as a hub. In TE cells, *FABP3* and *EPCAM*, crucial for metabolism regulation, were identified as the main hub genes. In conclusion, the EHMT1/2 inhibitor A366 mainly affects PRC2-related genes, which could be linked to changes in H3K27 methylation. Meanwhile, genes with increased expression are involved in a variety of signaling pathways and metabolic processes.

### Impact of HDAC class I inhibition on gene expression in EPI, PE and TE lineages

**Fig. 7:**
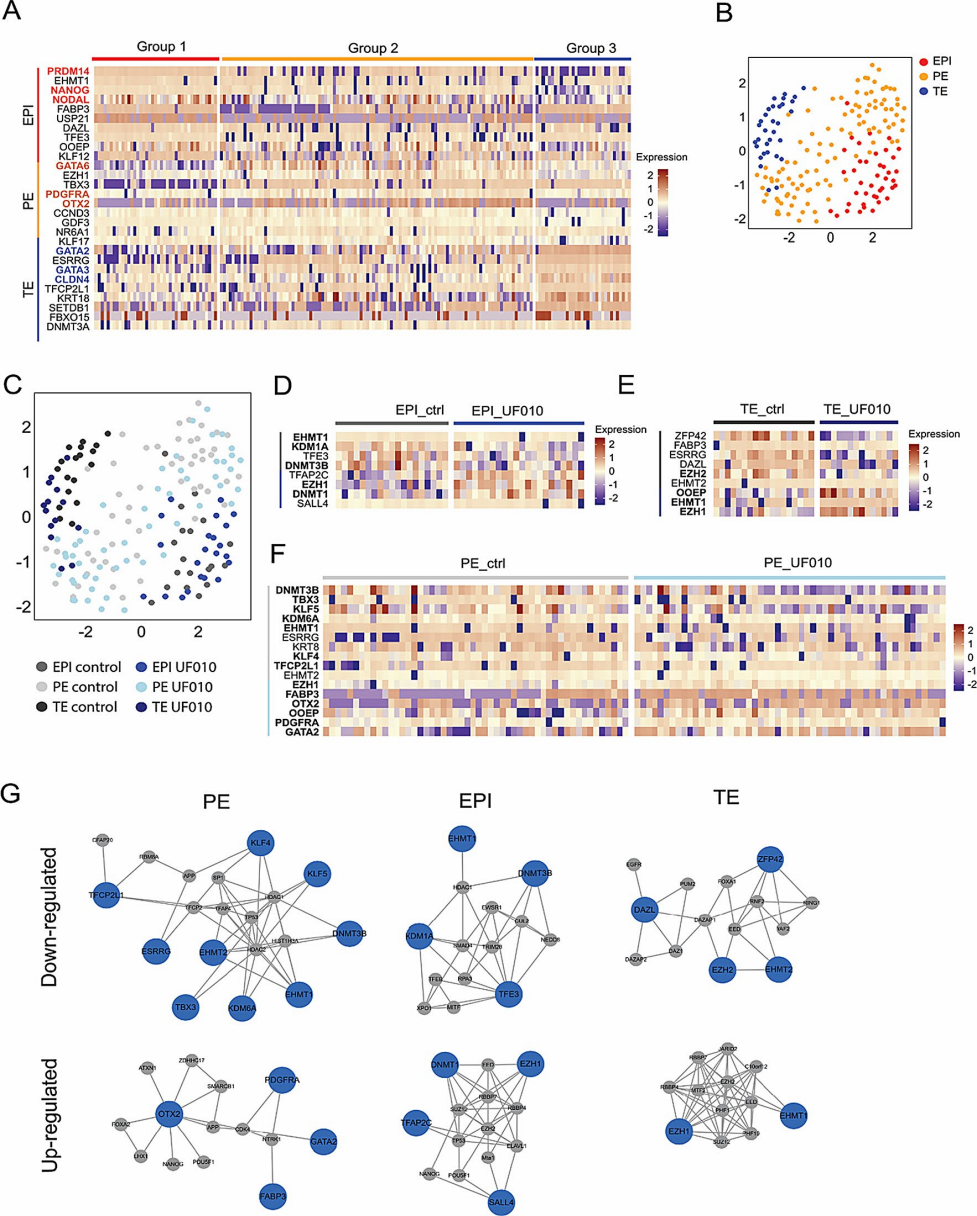
Effect of class I HDAC inhibition on the transcriptome of rabbit embryos. (**A**) Heatmap illustrating differentially expressed genes among EPI, PE and TE groups. (**B**) Two-dimensional UMAP representation of samples, color-coded according to embryonic lineage. (**C**) Two-dimensional UMAP representation of samples color-coded by both lineage and culture condition. (**D-F**) Heatmap of differentially expressed genes between control samples and those treated with 2 µM UF010 for 48 h within the EPI (**D**), TE (**E**), and PE (**F**) lineage. In panels **A**,** D**, **E** and **F**, expression is shown in log2Ex scaled and log-normalized format. (**G**) The protein-protein interaction (PPI) network displays differentially expressed genes (DEGs) as seed nodes in blue and their top 10 ranking neighbors in grey for each cell lineage. Statistically significant correlations between DEGs are depicted as edges (grey lines). This analysis was conducted separately for downregulated and upregulated genes

Single-cell expression analysis of the selected genes was conducted using Biomark RT-qPCR on UF010-treated embryos and untreated controls, with the same approach as used for A366 inhibitor. After data filtration, 89 samples from each group were analyzed, and control samples were compared to UF010-treated samples. K-means clustering was applied to categorize the samples into the three embryonic lineages, confirmed by lineage-specific gene expression: *NANOG*,* PRDM14*, and *NODAL* for EPI; *GATA6*, *OTX2*, and *PDGFRA* for PE; and *GATA2*, *GATA3*, and *CLDN4* for TE (Fig. 7A). UMAP dimension reduction was used to visualize the data, on which we identified the three distinct clusters representing EPI, PE, and TE, respectively (Fig. 7B). When overlaid with cell origins (UF010-treated versus control), minimal segregation was observed (Fig. 7C).

Differential analysis indicated that in EPI cells, UF010 treatment led to the upregulation of *EZH1* and *DNMT1*, while genes encoding chromatin regulators such as *KDM1A*, *EHMT1*, and *DNMT3B* were downregulated (Fig. 7D and S5). In TE cells, UF010 treatment resulted in upregulation of *OOEP*, *EZH1*, and *EHMT1*, and downregulation of *EZH2* (Fig. 7E and S5). In PE cells, UF010 treatment increased expression of PE markers *OTX2* and *PDGFRA*, along with TE markers *FABP3*, and *GATA2*, while decreasing expression of chromatin regulator genes (*DNMT3B*, *EHMT1*, *KDM6A*) and pluripotency genes (*TBX3*, *KLF5*, *KLF4*, *OOEP*, *TFCP2L1*) (Fig. 7F and S5). Notably, SOX2 expression was significantly increased in both PE and EPI cells (Fig. S5). These findings indicate that UF010 treatment impacts gene regulation across the embryonic lineages, influencing developmental pathways.

We performed a PPI network analysis, concentrating on the top 10 neighbors for each cell type (EPI, PE and TE) following UF010 treatment. The analysis revealed that in the networks calculated with genes whose expression was down-regulated in the PE and EPI, the *HDAC1* and *HDAC2* genes were central hubs, connecting to other genes with reduced expression (Fig. 7G**)**. This pattern suggests a feedback loop between the protein targeted by UF010 and the genes encoding it. *EHMT1/2* genes were also downregulated. Additionally, transcription factor *TFE3* in EPI cells, and RNA- and DNA-binding proteins *DAZL* and *ZFP42*/*REX1* in TE cells were identified as major hubs. In PE cells, *OTX2* stood out as a significant hub gene. For genes with increased expression, in both TE and EPI cells, *EZH1*, a core component of the PRC2 complex, was recognized as a hub gene. *SALL4*, a regulator of pluripotency, was also prominent in these cells. Overall, the treatment with the HDAC Class I inhibitor UF010 primarily impacts genes related to the PRC2 complex, highlighting the interplay between H3K9 modifications and H3K27 methylation. The primary genes that were upregulated are involved in various epigenetic modifications and the regulation of stem cell properties.

## Discussion and conclusions

In this study, we explored the roles of post-translational modifications of histones in the lineage specification of rabbit blastocysts. We observed dynamic changes in H3K9 modifications during early embryonic development, with H3K9me2/3 levels decreasing during blastocyst formation and cavitation, especially in inner cell mass (ICM) cells. In contrast, H3K9ac was prevalent in the early blastocyst stages, particularly in ICM cells, and decreased as cells transitioned into the epiblast (EPI). These alterations were coupled with differential gene expression of chromatin regulators such as EHMT1, EHMT2, SETDB1 (histone methyltransferases), and HDACs (histone deacetylases), suggesting that these histone modifications play pivotal roles in regulating lineage specification during blastocyst development. Our data show that partial inhibition of H3K9me2/3, using the specific EHMT1/2 inhibitor A366, disrupts primitive endoderm (PE) segregation and diminishes its formation. Conversely, UF010 treatment, which significantly enhances H3K9ac levels as well as H3K27ac and H4ac, promotes EPI expansion at the expense of PE. These findings emphasize the crucial role of histone modifications in balancing cell fate decisions during early development.

Moreover, our results support the concept of an “epigenetic clean state”, or *tabula rasa*, in the early epiblast. This state is maintained both passively, through the low expression of enzymes that add repressive marks, and actively, through the high expression of enzymes that remove these marks and add permissive ones. Notably, many genes that are highly expressed in the ICM of early blastocysts, which encode proteins involved in chromatin organization, are also highly expressed in naive pluripotent stem cells (PSCs) derived from macaque and mouse blastocysts [27]. This suggests a highly conserved mechanism of heterochromatin regulation across mammalian species.

Despite targeting enzymes associated with heterochromatin formation, the inhibitors used in our study produced markedly different effects. Notably, inhibition of class I HDACs appears to enhance epiblast cell development, representing the first demonstration of HDAC inhibitors positively impacting embryonic pluripotent cells within developing embryos. This finding contrasts with earlier studies in mice, where suppression of Hdac1 and 2 was detrimental, notably reducing blastocyst formation by increasing apoptosis [34, 35], and where HDAC inhibition reprograms ESC to TSC [36]. The distinct outcome observed in our study may be attributed to species differences (rabbit versus mouse) and the specific timing of UF010 administration, just before blastocyst formation. This strategic timing likely accounts for the variations observed in earlier studies. Supporting this hypothesis, prior research has demonstrated that HDAC inhibitors facilitate the transition of pluripotent stem cells (PSCs) from the primed to the naive state in both mice and humans [21, 22]. Notably, Collier et al. showed that HDAC2-specific inhibition improves naive PSC reprogramming efficiency [37]. Our use of UF010, which selectively targets class I HDACs, including HDAC2, could explain the observed beneficial impact on the development of naïve pluripotent embryonic cells.

In comparing UF010-treated and untreated embryos, we observed significant differences in the expression of many chromatin regulators, suggesting that chromatin remodeling is occurring in the UF010-treated cells. One notable consequence of this treatment is the increased number of cells expressing SOX2, a marker of pluripotency. This increase in SOX2 expression may blur the distinction between EPI and PE, suggesting that elevated SOX2 could prevent proper SOX17 protein expression, thereby disrupting PE differentiation. Strikingly, despite the increased SOX2 expression, we were still able to identify cells from all three lineages in the single-cell RT-qPCR analysis. This suggests that SOX2 expression alone is insufficient to completely block non-epiblast cell-fate. In addition, perturbations in cell fate may rise from altered expression of genes that regulate the chromatin state. It would be interesting to investigate whether UF010 treatment affects early embryonic lineage plasticity in future studies.

A particularly interesting finding is the higher expression of the *OOEP* gene in cells from UF010-treated embryos. *OOEP* belongs to the *KHDC1* locus family of genes, which is known to be consistently overexpressed in the naive state of cells, regardless of the experimental model–whether in vitro or in vivo–as reported in several studies [38–43]. Despite this consistent expression pattern in naive cells, the specific roles of these genes, including *OOEP*, in regulating naive pluripotency remain poorly understood.

We demonstrate that inhibiting EHMT1 and EHMT2 with A366 slows the development of rabbit blastocysts and disrupts PE formation. In mice, knockdown of the *Ehmt1* gene leads to slower development and disrupts the expression of the *Oct4*/*Pou5f1*, *Sox2*, and *Nanog* genes [44, 45]. Furthermore, maternal knockout of the *Ehmt2* gene results in a reduction in the number of Sox17^+^ cells and a decrease in the total cell count in the blastocyst [45]. This developmental delay can be attributed, at least in part, to an increase in apoptosis resulting from DNA damage [44, 45]. The authors also observed a reduction in the number of cells in the ICM, due to both a decrease in Sox17^+^ cells and an increase in Sox17^−^/Sox2^−^ cells. Our results are consistent with those observations in mice. The growth retardation seen in A366-treated rabbit embryos could be explained by transcriptomic changes induced by the inhibition of H3K9 methylation in two ways: 1 - Loss of H3K9 methylation leads to the accumulation of DNA damage due to the de-repression of repeated elements of the genome, as observed in mice [44]. This accumulation of DNA damage increases apoptosis, thereby reducing embryo size. 2 - Loss of H3K9 methylation disrupts lineage allocation, resulting in cells that are no longer able to specify themselves correctly in the PE or epiblast. It has been shown that in the mouse pre-implantation embryo, cells failing to commit to the PE or EPI undergo apoptosis through cell competition [46].

In conclusion, we demonstrated that inhibitors of heterochromatin-promoting enzymes increase the expression of pluripotency genes in rabbit embryos, inhibit PE formation, and promote EPI cell proliferation. Manipulating heterochromatin could thus be a potent method for controlling naive pluripotency. Testing the effects of UF010 and A366 on rabbit ESCs is now under investigation, as this approach may facilitate the propagation of naive rabbit ESCs.

## Electronic supplementary material

Below is the link to the electronic supplementary material.

Supplementary Material 1

Supplementary Material 2

Supplementary Material 3

Supplementary Material 4

## Data Availability

Biomark single-cell data are available in Tables S2 and S3.10x single-cell RNAseq datasets are deposited in NCBI GEO under the accession number GSE180048.

## Abbreviations

EHMT: euchromatin histone methyl transferase
EPI: epiblast
H3K9ac: histone H3 lysine 9 acetylated
H3K9me2: histone H3 lysine 9 di-methylated
H3K9me3: histone H3 lysine 9 tri-methylated
HAT: histone acetyl transferase
HDAC: histone deacetylase
ICM: inner cell mass
KDM: lysine demethylase
PE: primitive endoderm
TE: trophectoderm
