# Supplementary material for: H3K9 post-translational modifications regulate epiblast/primitive endoderm specification in rabbit blastocysts
Source: Epigenetics Chromatin. 2025 Jan 13;18:2. doi: 10.1186/s13072-025-00568-8 (PMC11727677; doi:10.1186/s13072-025-00568-8)
Supplement: Supplementary file 2 — Supplementary Material 2 [file 13072_2025_568_MOESM2_ESM.docx]

**SUPPLEMENTARY DATA**

**H3K9 post-translational modifications regulate epiblast/primitive endoderm specification in rabbit blastocysts /** BOUCHEREAU, PHAM, SAMRUAN et al.

**
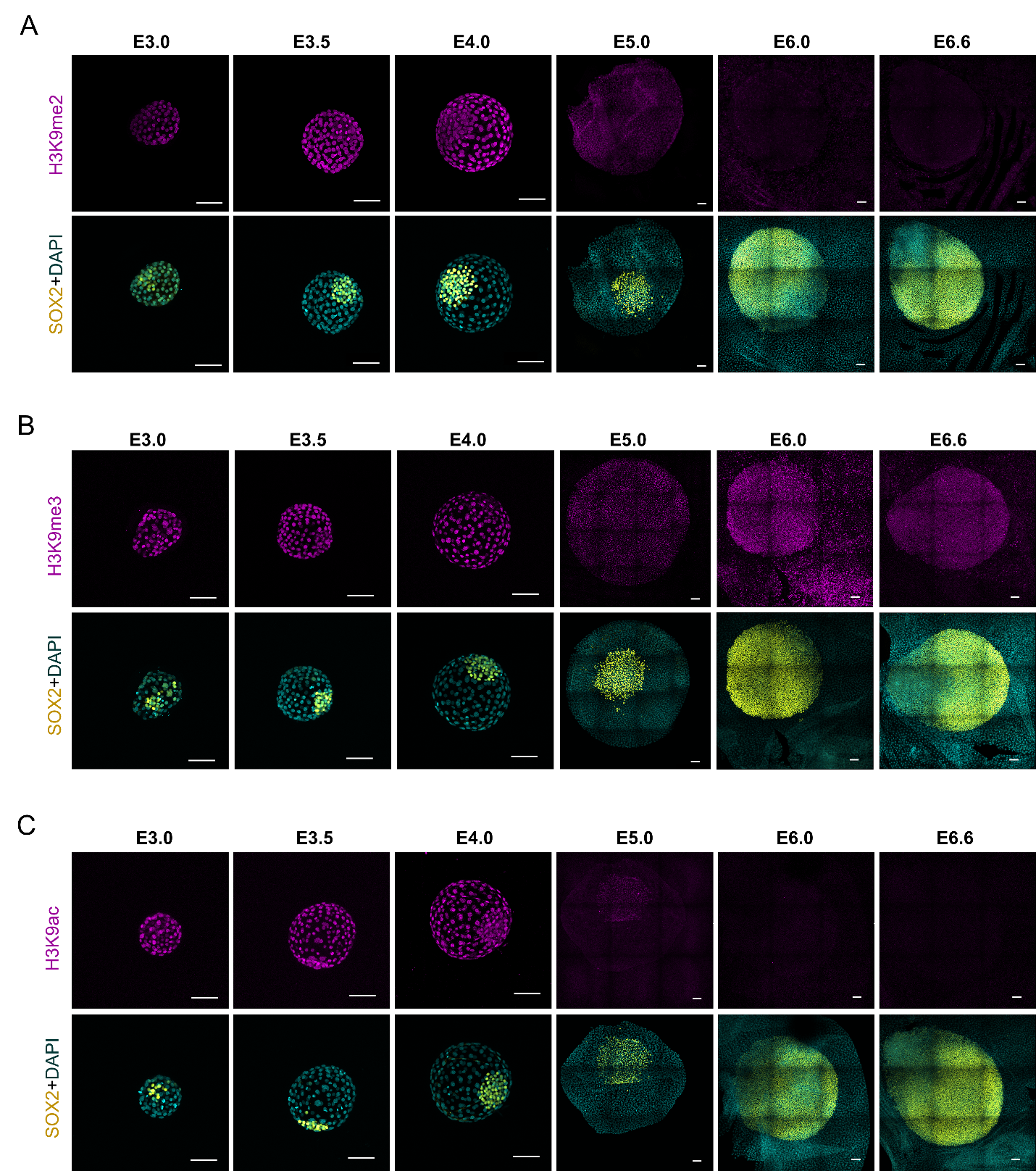
**

**Figure S1: Co-detection of SOX2 and** **H3K9 methylation/acetylation in rabbit embryos**. Immunofluorescent detection of H3K9me2 (A), H3K9me3 (B), and H3K9ac (C) was performed on embryos at stages E3.0, E3.5, E4.0, E5.0, E6.0, and E6.6. Each histone mark is visualized in magenta, with co-detection of SOX2 in yellow and DNA counterstaining using DAPI in cyan. Three experiments were conducted for each histone mark, with 3 to 10 embryos analyzed per stage. Scale bars: 100µm.

**
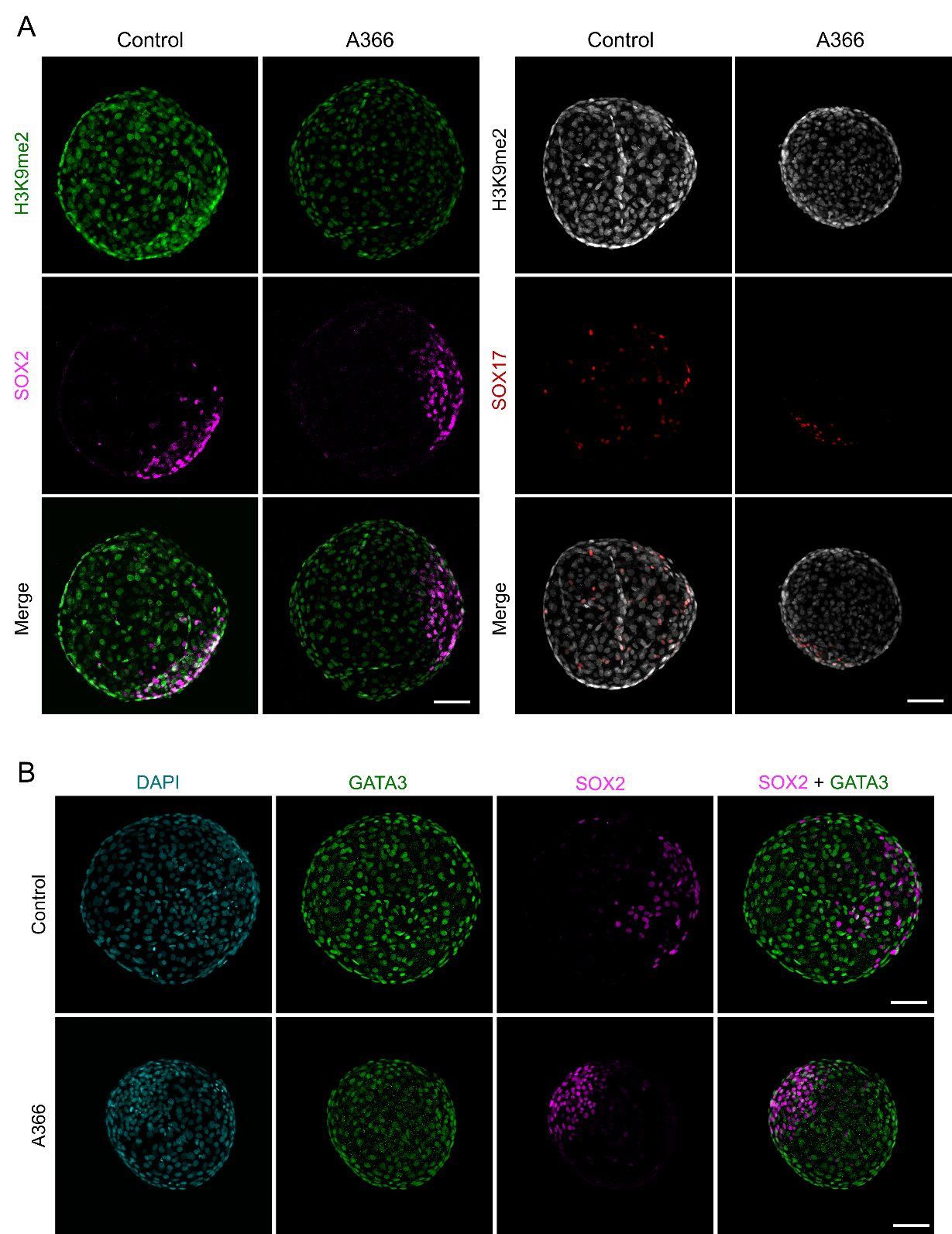
**

**Figure S2: Immunolabelling of rabbit embryos treated with or without A366**

1. Immunolabeling for H3K9me2 mark along with SOX2 or SOX17 (n=3 independent experiments). (B) Immunolabeling for GATA3 along with SOX2 showing no overlap between these stainings. DNA was counterstained with DAPI (n = 12 for controls and 11 for A366-treated embryos, 2 independent experiments). Scale bars: 100µm.

**
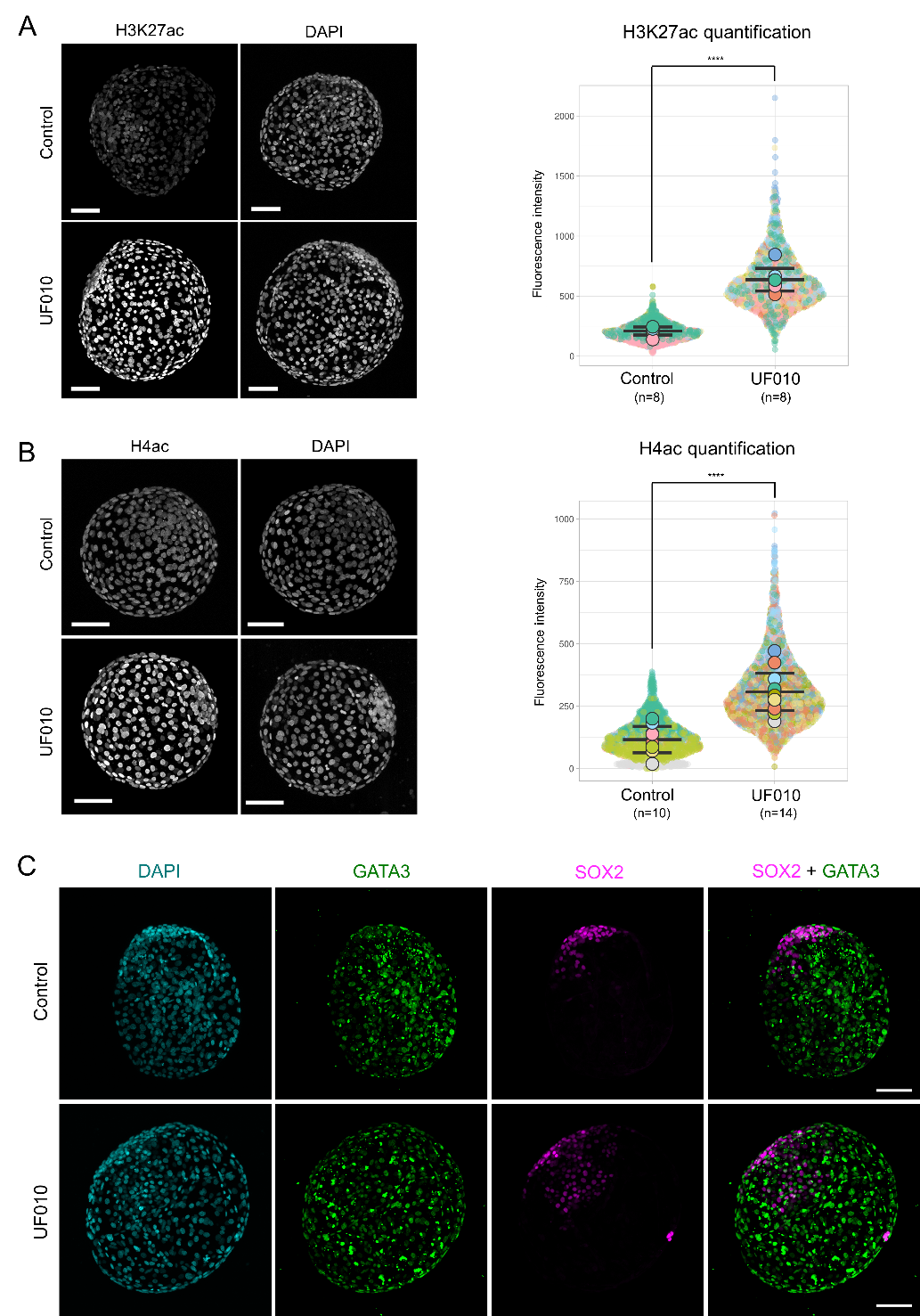
**

**Figure S3: Effect of UF010 on rabbit embryos.**

Immunolabeling of acetylated Histone 3 lysine 27 (A) and acetylated Histone 4 (B) was conducted in two different experiments for each modification, on embryos treated with or without UF010. DNA was counterstained with DAPI. Scale bars: 100µm. For each condition, fluorescence intensity was quantified across whole embryos. In the corresponding violin plots, each color represents a single embryo, with the mean intensity per embryo indicated as a larger dot. ****, p < 0.0001. (C) Immunolabeling for GATA3 along with SOX2 showing no overlap between these stainings. DNA was counterstained with DAPI (n = 5 for controls and 5 for UFO-treated embryos, 1 experiment). Scale bars: 100µm.

**
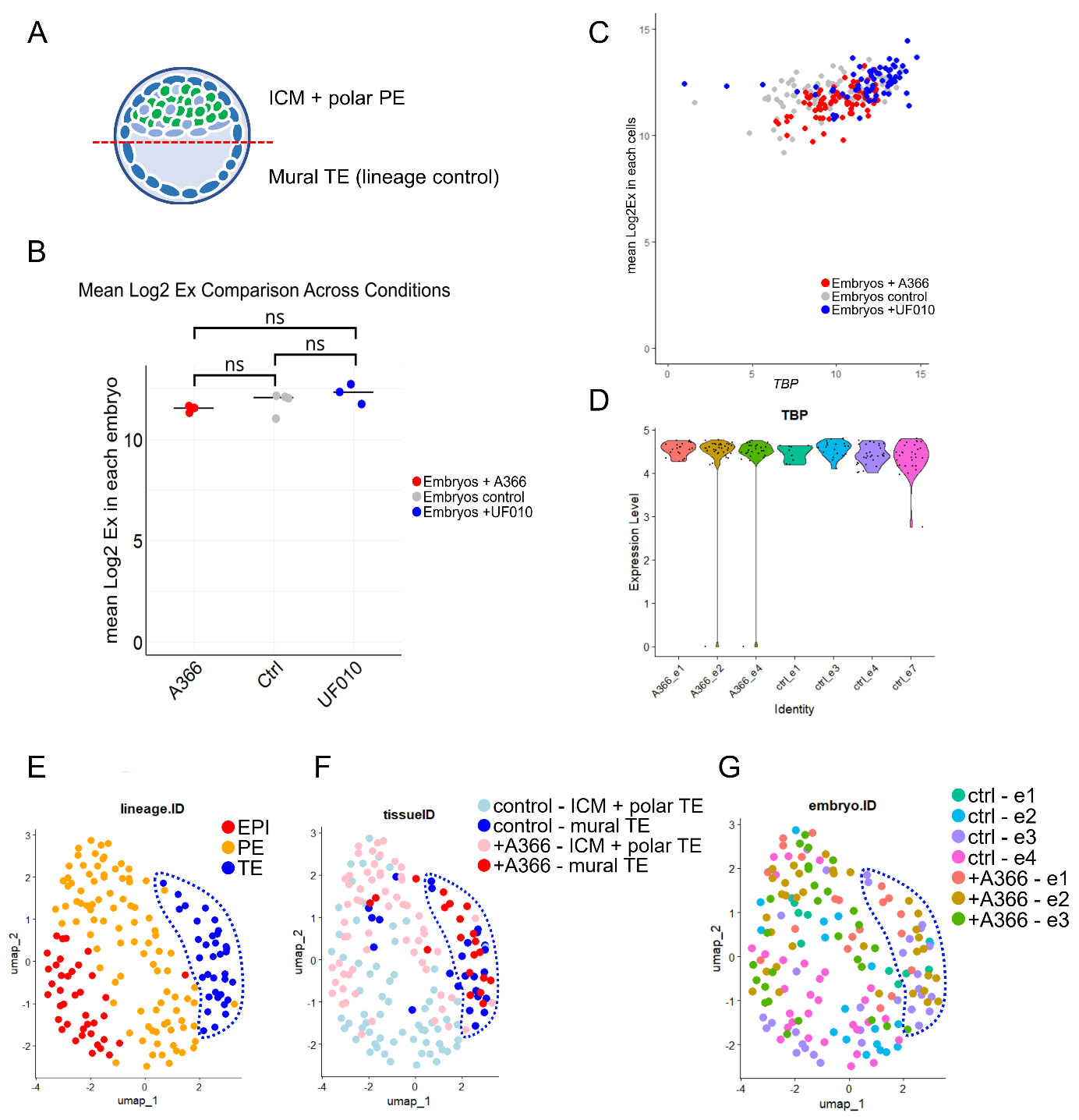
**

**Figure S4: Single-cell qPCR controls.**

(A) Schematic representation of embryo dissection prior to single-cell dissociation and collection. Embryos were mechanically divided in two parts. Most harvested cells were derived from the polar side of the embryos (polar TE + ICM), while some cells from the opposite side (mural TE) were collected as TE positive controls. (B) Mean Log2Ex values of all target genes per cell for each embryo, illustrating the absence of batch effects. Each dot represents a single embryo. (C) Scatter plot showing the expression of the *TBP* housekeeping gene in log2Ex values versus the mean log2Ex of all target genes, indicating consistent gene expression levels across embryos. Each dot represents a single cell. (D) Single-cell expression of *TBP* in A366-treated and untreated embryos after scaling and normalization using Seurat software. Each point represents one cell. (E-G) UMAP visualization of single-cell qPCR results from A366-treated and untreated embryos after scaling and normalization. Cells were either colored by their assigned lineage based on biocomputational analysis (E), by treatment status and cell origin, as determined during dissection (F) or by embryos (G). Notably, mural TE control cells colocalize with the TE-assigned population and cells were broadly distributed in the UMAP space with no ‘embryo-effect’.

**
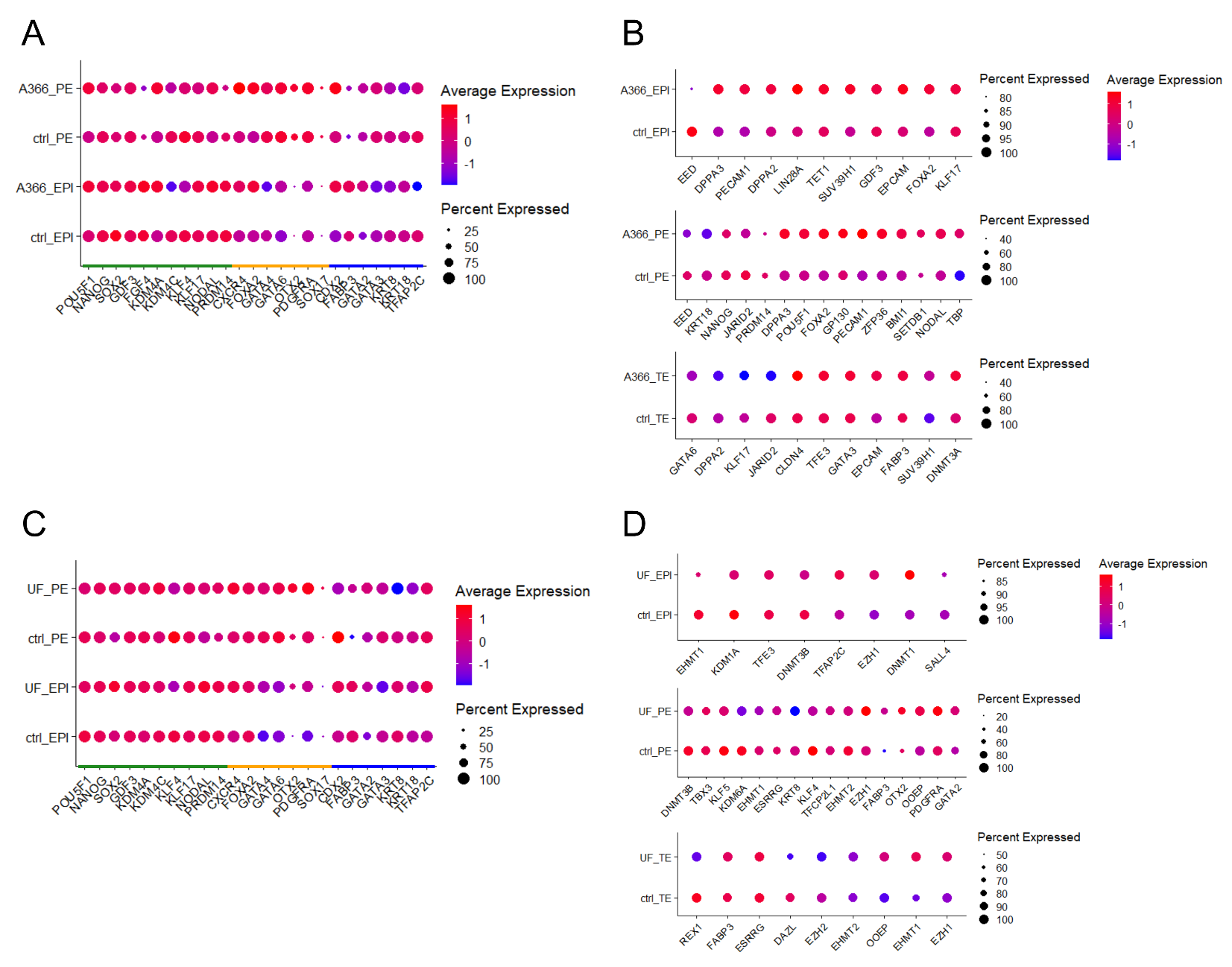
**

**Figure S5: Differential analysis between control and treated embryos.**

(A) Dot plot showing the average expression of epiblast, primitive endoderm and trophectoderm markers in the three lineages in control and A366-treated embryos. (B) Differentially expressed genes between control or A366-treated embryos in epiblast cells (first panel), primitive endoderm (second panel), and trophectoderm (third panel). (C) Dot plot showing the average expression of epiblast, primitive endoderm, and trophectoderm markers in the three lineages in control and UF010-treated embryos. (D) Differentially expressed genes between control and UF010-treated embryos in epiblast cells (first panel), primitive endoderm (second panel), and trophectoderm (third panel).
